# The effect of depression management on diabetes and hypertension outcomes in low- and middle-income countries: a systematic review protocol

**DOI:** 10.1186/s13643-018-0896-1

**Published:** 2018-12-05

**Authors:** Michael Udedi, Brian Pence, Felix Kauye, Adamson S. Muula

**Affiliations:** 1 College of Medicine, University of Malawi, P/Bag 360, Chichiri, Blantyre, 3 Malawi; 2 Department of Epidemiology, University of North Carolina-Chapel Hill, McGavran-Greenberg, 2103C Campus Box 7435, UNC-Chapel Hill, Chapel Hill, NC 27599-7435 USA

**Keywords:** Depression, Non-communicable diseases, Diabetes, Hypertension, Outcomes, Low- and middle-income countries, Systematic review, Protocol

## Abstract

**Background:**

Depression and non-communicable diseases (NCDs) account for a growing burden on health systems in low- and middle-income countries (LMICs). Depression is generally associated with the outcomes of NCDs and is an important barrier to consistent NCD care management. There is great need to understand the efficacy of interventions to treat depression for people with NCDs, but there is a paucity of evidence of the efficacy of the interventions in LMICs. Therefore, the broad objective of this review is to systematically review the literature on the effectiveness of depression management among patients with diabetes and hypertension to improve outcomes.

**Methods:**

This is a systematic review to assess the evidence of the effect of depression management in diabetic and hypertensive patients on diabetes and hypertension outcomes in LMICs. Two independent reviewers will search articles on PubMed, EMBASE, PsycINFO, and Global Index Medicus. Two reviewers will then screen the articles independently based on predefined criteria. We will use standard methods as recommended by the Cochrane Collaboration of assessing quality of evidence and publish our report using the PRISMA guidelines.

**Discussion:**

The findings from this review will provide evidence to be used in guiding practice and policy on how to integrate depression management in diabetes and hypertension clinics.

**Systematic review registration:**

PROSPERO CRD42017068257

## Background

Non-communicable diseases (NCDs) are a growing burden on the health systems in low- and middle-income countries (LMICs) [1, 2]. Studies show that the comorbidity of depression and chronic diseases such as diabetes is common in LMICs and they are significant causes of morbidity, disability, and mortality [3, 4]. Depression is a mood disorder characterized by changes in ones feelings (e.g., feels sad or empty), physical status (e.g., loss of energy), thought process (e.g., diminished ability to think or concentrate, or more indecisiveness), and behavior (e.g., thoughts of death or suicide) [5]. Depression is estimated to affect 350 million people worldwide [6, 7]. The prevalence of comorbidity of physical NCDs, such as diabetes and hypertension, and depression is increasing. The 12-month prevalence of comorbid depression with NCDs worldwide is between 9.3% and 23.0% of people [8, 9]. Depression affects one in three people with hypertension [6] while one in four patients with diabetes have depression [10, 11]. Modifiable risk factors for NCDs are exacerbated by poor mental health, and on the other hand, NCDs are a risk factor for depression [12]. Evidence indicates a bidirectional relationship between depression and individual chronic diseases [10]; for instance, depression increases the risk of the development of type 2 diabetes and subsequent risks of hyperglycemia [11]. Furthermore, a diagnosis of type 2 diabetes increases the risk of depression and can contribute to a more severe course of depression [11]. Some studies have found non-adherence to prescribed medication among cardiovascular diseases (CVD) patients with depressive symptoms [13, 14]. Non-adherence to medication has been suggested to be a contributor to the increased morbidity and mortality seen in CVD and comorbid mental disorders [15]. Many studies that examined the prevalence of depression among patients with diabetes mellitus and hypertension recommend including routine screening of depression in patients with diabetes or hypertension and referral for appropriate management [16–19].

A systematic review by Mendenhall et al. showed that one in three people with diabetes in LMICs has co-morbid depression which is suggested to be higher than in higher income countries [2]. As such, the review concluded that there is need for integrating mental health care into diabetes care within LMIC health systems. Furthermore, the review recommended the need for research around the cost of oversight of mental health problems among people with chronic illness in LMICs [2]. The review highlighted that treating depression among people with diabetes is cost-effective in high-income countries (HICs) [2]. The observation made in the Mendenhall review confirms findings of another systematic review of comorbid depression and diabetes in the USA (by Atlantis et al.) which suggested that collaborative care for depression significantly improves both depression and glycaemia outcomes [20]. The systematic reviews from HICs indicate that collaborative care is more effective than usual care for improving depression outcomes in people with comorbid diabetes [20–22]; however, there is paucity of data on the effect of treating depression among patients with diabetes and hypertension in LMICs. The National Institute for Health and Care Excellence (NICE) guidelines for depression in adults with a chronic physical health problem, such as diabetes and hypertension, recommend collaborative care [23]. Accordingly, there is great need to understand the efficacy of interventions to treat depression for people with chronic disease since the Mendenhall review did not address the efficacy of interventions. The Atlantis review looked at intervention efficacy but did not identify LMIC studies for inclusion. Therefore, our systematic review will assess the evidence of the effect of management of depression in diabetic and hypertensive patients on diabetes and hypertension outcomes in LMICs. Our review also aims to identify efficacy LMIC studies that may have been missed by previous studies by searching the Global Health database and using search terms to identify LMIC studies. The systematic review will inform practice and policy on the need to integrate depression management in the management of patients with diabetes and hypertension.

## Methods

Our objective is to identify studies examining the effect of depression management on diabetes and hypertension outcomes. We will use the Cochrane Collaboration Handbook [24] as the systematic review guide in conducting the systematic review. We will utilize the Preferred Reporting Items of Systematic Reviews and Meta-Analyses Protocol (PRISMA) [25, 26] guidelines in reporting the review. The protocol has been registered on the PROSPERO database (Ref: CRD42017068257) and will follow the Preferred Reporting Items of Systematic Reviews and Meta-Analyses Protocol (PRISMA-P) guidelines [27].

### Data sources and searches

We will search four databases from 1990 to present: PubMed, EMBASE, PsycINFO, and Global Index Medicus. We will include all studies from 1990 because we anticipated that adequate number of studies would have been published thereafter to meaningfully examine the effectiveness of depression management on NCDs in LMICs. The search strategy will include the search concepts (1) depression, (2) interventions for depression management, (3) diabetes or glycemic control or hypertension or blood pressure control, and (4) LMICs. Appropriate controlled vocabulary words will be tailored to each database. Some of the search terms are based on existing reviews [2, 6, 20, 28]. We have developed an electronic search strategy of combined search terms for one database as shown in Table 1. We will also search reference lists of all studies that will meet our inclusion criteria and relevant systematic reviews for additional potentially eligible primary studies. Authors of included reviews will be contacted to clarify reported published information and to seek unpublished results. We will perform a forward citation search to identify additional studies. Appendices for all strategies used, including a list of sources screened and relevant reviews or primary studies reviewed, will be provided. We will exclude non-English articles during the full-text screening stage as resources are not available to translate the data. Any potentially relevant non-English articles will be listed in review to indicate further available evidence.

**Table 1:** Search strategy

Database	Search terms
PubMed	((((africa[MeSH Terms]) OR (((Africa[Title/Abstract] OR Asia[Title/Abstract] OR “low-income country”[Title/Abstract] OR “low-income countries”[Title/Abstract] OR “low” [Title/Abstract] AND “middle-income countries”[Title/Abstract] OR “middle-income country”[Title/Abstract] OR “middle income countries”[Title/Abstract] OR “Sub-Saharan Africa”[Title/Abstract] OR Afghanistan[Title/Abstract] OR Benin[Title/Abstract] OR “Burkina Faso”[Title/Abstract] OR Burundi[Title/Abstract] OR “Central African Republic”[Title/Abstract] OR Chad[Title/Abstract] OR Comoros[Title/Abstract] OR Congo[Title/Abstract] OR Eritrea[Title/Abstract] OR Ethiopia[Title/Abstract] OR Gambia[Title/Abstract] OR guinea[Title/Abstract] OR “Guinea-Bissau”[Title/Abstract] OR Haiti[Title/Abstract] OR “North Korea”[Title/Abstract] OR “Democratic People’s Republic of Korea”[Title/Abstract] OR Liberia[Title/Abstract] OR Madagascar[Title/Abstract] OR Malawi[Title/Abstract] OR Mali[Title/Abstract] OR Mozambique[Title/Abstract] OR Nepal[Title/Abstract] OR Niger[Title/Abstract] OR Rwanda[Title/Abstract] OR Senegal[Title/Abstract] OR “Sierra Leone”[Title/Abstract] OR Somalia[Title/Abstract] OR “South Sudan”[Title/Abstract] OR Tanzania[Title/Abstract] OR Togo[Title/Abstract] OR Uganda[Title/Abstract] OR Zimbabwe [Title/Abstract] OR Armenia[Title/Abstract] OR Bangladesh[Title/Abstract] OR Bhutan[Title/Abstract] OR Bolivia[Title/Abstract] OR “Cabo Verde”[Title/Abstract] OR “Cape Verde”[Title/Abstract] OR Cambodia[Title/Abstract] OR Cameroon[Title/Abstract] OR Congo[Title/Abstract] OR “Cote D’Ivoire”[Title/Abstract] OR Djibouti[Title/Abstract] OR Egypt[Title/Abstract] OR “el Salvador”[Title/Abstract] OR Ghana[Title/Abstract] OR Guatemala[Title/Abstract] OR Honduras[Title/Abstract] OR India[Title/Abstract] OR Indonesia[Title/Abstract] OR Kenya[Title/Abstract] OR Kiribati[Title/Abstract] OR Kosovo[Title/Abstract] OR Kyrgyz[Title/Abstract] OR Kyrgyzstan[Title/Abstract] OR Lao[Title/Abstract] OR Laos[Title/Abstract] OR Lesotho[Title/Abstract] OR Mauritania[Title/Abstract] OR Micronesia[Title/Abstract] OR Moldova[Title/Abstract] OR Mongolia[Title/Abstract] OR Morocco[Title/Abstract] OR Myanmar[Title/Abstract] OR Nicaragua[Title/Abstract] OR Nigeria[Title/Abstract] OR Pakistan[Title/Abstract] OR “Papua New Guinea”[Title/Abstract] OR Philippines[Title/Abstract] OR Samoa “Sao Tome”[Title/Abstract] OR Principe[Title/Abstract] OR “Solomon Islands”[Title/Abstract] OR “Sri Lanka”[Title/Abstract] OR Sudan[Title/Abstract] OR Swaziland[Title/Abstract] OR Syria[Title/Abstract] OR “Syrian Arab Republic”[Title/Abstract] OR Tajikistan[Title/Abstract] OR Timor[Title/Abstract] OR Tonga[Title/Abstract] OR Tunisia[Title/Abstract] OR Ukraine[Title/Abstract] OR Uzbekistan[Title/Abstract] OR Vanuatu[Title/Abstract] OR Vietnam[Title/Abstract] OR “West Bank”[Title/Abstract] OR Gaza[Title/Abstract] OR Yemen[Title/Abstract] OR Zambia [Title/Abstract] OR “developing countries”[MeSH Terms]))) AND (((diabetes[Title/Abstract] OR diabetic[Title/Abstract] OR hypertension[Title/Abstract] OR “blood pressure”[Title/Abstract] OR hypertensive[Title/Abstract])) OR “hypertension”[MeSH Terms] OR “diabetes mellitus”[MeSH Terms])) AND (((depression[Title/Abstract] OR depressed[Title/Abstract] OR depressive[Title/Abstract])) OR “depressive disorder” [MeSH Terms]) OR depression[MeSH Terms] AND (“screening” [Title/Abstract] OR “Mass Screening” [MeSH Terms]) AND (“CBT” [Title/Abstract] OR “Antidepressant Medication” [Title/Abstract] OR “Psychological Intervention” [Title/Abstract])

### Eligibility criteria

Any studies that examined the outcomes of an intervention for depression management on diabetes or hypertension outcomes (HbA1c, fasting blood glucose, depression scores, and blood pressure (BP) measurements) for patients with diabetes mellitus or hypertension will be eligible for inclusion. The minimum and maximum time periods for follow-up of diabetes and hypertension health outcomes will be 3 and 12 months respectively.

Depression is a mood disorder characterized by changes in ones feelings (e.g., feels sad or empty), physical status (e.g., loss of energy), thought process (e.g., diminished ability to think or concentrate, or more indecisiveness), and behavior (e.g., thoughts of death or suicide) [5].

Hypertension is defined as systolic BP level of ≥ 140 mmHg and diastolic BP of ≥ 90 mmHg [29]. The diagnostic of hypertension is made when the average of two or more diastolic BP measurements on at least two subsequent visits is ≥ 90 mmHg or when the average of multiple systolic BP readings on two or more subsequent visits is consistently ≥ 140 mmHg.

Diabetes is defined as a group of metabolic diseases characterized by hyperglycemia resulting from defects in insulin secretion, insulin action, or both [30]. The criteria for diagnosis of diabetes include a fasting plasma glucose (FPG) level > 126 mg/dl (7.0 mmol/l) or a casual plasma glucose > 200 mg/dl (11.1 mmol/l) or HbA1C test with a threshold of ≥ 6.5% meets the threshold for the diagnosis of diabetes [30]. We will only include type 2 diabetes in our review; thus, type 1 diabetes and gestational diabetes will be excluded.

The following PICOS describes the review inclusion criteria and search strategy concepts:

*Population*: Adults (18 years or older) suffering from diabetes or hypertension or both conditions.

*Intervention*: Depression management interventions including screening strategy for depression, psychological intervention, or medication management.

*Comparator*: No routine screening or diagnosis for depression in patients with diabetes or hypertension.

*Outcome*: Diagnosis of depression; HBA1c, fasting blood glucose, blood pressure control, and depression scores.

*Study design*: Randomized control trials and quasi-experimental and observational studies will be eligible for inclusion because of paucity of data from LMICs.

*Setting*: Primary and secondary health care service delivery in LMIC. We only include studies conducted in the low-income countries and the lower-middle income countries as defined by the World Bank [31].

We will exclude studies involving patients less than 18 years old and those with additional co-occurring medical conditions.

### Study selection

We will download all titles and abstracts retrieved by electronic searching to Endnote and remove duplicates. Two reviewers [MU and MN] will independently screen the titles and abstracts for inclusion. We will retrieve the full-text study reports/publication, and two reviewers [MU and MN] will independently screen the full text and identify studies for inclusion and identify and record reasons for exclusion of the ineligible studies. We will resolve any disagreement through discussion or, if required, we will consult a third reviewer [MM] for consensus agreement. We will list studies that initially appeared to meet the inclusion criteria but that we later excluded. We will collate multiple reports of the same study so that each study rather than each report is the unit of interest in the review. We will also provide any information we can obtain about ongoing studies. We will record the selection process in sufficient detail to complete a PRISMA flow diagram.

### Data extraction and management

We will use the Cochrane EPOC (Effective Practice and Organisation of Care) data collection form and adapt it for our study characteristics. We will pilot the form and adjust accordingly to suit the required study characteristics. We will include studies reported in full. Abstracts will be excluded if they do not have sufficient data. Two reviewers [MU and MN] will independently extract the following study characteristics from the selected studies: study design, setting, and number of participants; intervention characteristics (type of intervention, intensity of the intervention, content of the intervention, and where intervention was given); and comparison group and outcomes (primary outcomes: glycated hemoglobin (HBA1c), blood pressure control, and depression scores).

### Risk of bias assessment

Two reviewers [MU and MN] will independently assess risk of bias for each study using the criteria outlined in the Cochrane Handbook for Systematic Reviews of Interventions [24]. Any disagreement will be resolved by discussion or by involving a third reviewer [MM]. We will assess the risk of bias according to the following domains: (1) random sequence generation, (2) allocation concealment, (3) blinding of participants and personnel, (4) blinding of outcome assessment, (5) incomplete outcome data, (6) selective outcome reporting, (7) baseline outcomes measurement, (8) baseline characteristics, and (9) other bias. We will judge each potential source of bias as high, low, or unclear and provide a quote from the study report together with a justification for our judgment in the “risk of bias” table. We will summarize the “risk of bias” judgements across different studies for each of the domains listed. We will consider blinding separately for different key outcomes where necessary. Where information on risk of bias relates to unpublished data, we will note this in the “risk of bias” table. We will not exclude studies on the grounds of their risk of bias, but will clearly report the risk of bias when presenting the results of the studies. When considering treatment effects, we will take into account the risk of bias for the studies that contribute to that outcome. We will use the Grading of Recommendations, Assessment, Development and Evaluation (GRADE) criteria in assessing the overall quality of evidence for specific quantitative outcomes.

## Data synthesis

We will follow data synthesis methods proposed in the PRISMA [25, 26] and Cochrane [24] publications. We will report studies using descriptive statistics. We will do both narrative synthesis and meta-analysis. In narrative synthesis, we will do structured synthesis using descriptive statistics and might use forest plot if necessary. A funnel plot will also be used to explore publication bias. In order to facilitate meta-analysis, prevalence data will be transformed using appropriate statistical methods. We will standardize data on the effects of depression management. We will assess methodological and statistical heterogeneity using *I*^2^. We will use Stata v.14 in the absence of statistically significant heterogeneity to pool and analyze the data using the random effects model in the event of multiple interventions or multiple outcomes. We will conduct sensitivity analyses on study quality and patient-related heterogeneity in order to explore the robustness of the results of the primary outcome.

## Discussion

We will report the findings from this review according to the PRISMA [25, 26]. The discussion will be drawn from findings of the synthesis. Practice- and policy-relevant aspects of applicability will be addressed in this section. The proposed systematic review will provide evidence which will be used in guiding practice and policy on how to integrate depression management in diabetes and hypertension care to improve patient outcomes.

## Potential limitations of review methods

These review findings may have a bias introduced by limiting the language to English-only articles published since 1990. The review team does not have the capacity to translate languages. The protocol will be revised to include studies published before 1990 if very few post-1990 are identified.

## Abbreviations

BP: Blood pressure
EPOC: Effective Practice and Organisation of Care
FPG: Fasting plasma glucose
GRADE: Grading of Recommendations, Assessment, Development and Evaluation
LMICs: Low- and middle-income countries
NCDs: Non-communicable diseases
PRISMA-P: Preferred Reporting Items of Systematic Reviews and Meta-Analyses Protocol
